# Construction and validation of a revised satisfaction index model for the Chinese urban and rural resident-based basic medical insurance scheme

**DOI:** 10.1186/s12911-022-02002-5

**Published:** 2022-10-03

**Authors:** Wenwei Cheng, Shiwen Wang, Xiaofang Liu, Yanyan Wu, Jin Cheng, Weichu Sun, Xiaofang Yan, Qi Wang, Liai Peng, Xiaoli Liu, Tingting Sha, Jingcheng Shi, Fang Yang

**Affiliations:** 1 Department of Medical Administration, The Third Xiangya Hospital, Central South University, Changsha, Hunan China; 2 Department of Epidemiology and Medical Statistics, Xiangya School of Public Health, Central South University, Xiangya Road 110, Changsha, 410078 Hunan China; 3 Department of Orthopedics, University of South China, Hengyang, Hunan China

**Keywords:** Satisfaction index model, Urban and rural resident-based basic medical insurance scheme, PLS-SEM, Reliability, Validity

## Abstract

**Background:**

Quality is the most important factor in satisfaction. However, the existing satisfaction index model of urban and rural resident-based basic medical insurance scheme (SIM_URRBMI) lacks the segmentation of perceived quality elements, it couldn’t provide a reference for quality improvement and satisfaction promotion. This study aims to construct a revised SIM_URRBMI that can accurately and detailly measure perceived quality and provide feasible and scientific suggestions for improving the satisfaction of urban and rural residents' basic medical insurance scheme (URRBMI) in China.

**Methods:**

Based on the theoretical framework of the American Customer Satisfaction Index, the elements of perceived quality were refined through literature review and expert consultation, and a pool of alternative measurement variables was formed. A three-stage randomized stratified cluster sampling was adopted. The main decision makers of URRBMI in the families of primary school students in 8 primary schools in Changsha were selected. Both the classic test theory and the item response theory were used for measurement variables selection. The reliability and validity of the model were tested by partial least squares (PLS)-related methods.

**Results:**

A total of 1909 respondents who had URRBMI for their children were investigated. The SIM_URRBMI1.0 consists of 11 latent variables and 28 measurement variables with good reliability and validity. Among the three explanatory variables of public satisfaction, perceived quality had the largest total effect (path coefficient) (0.737). The variable with the greatest effect among the five first-order latent variables on perceived quality was the quality of the medical insurance policy (0.472).

**Conclusions:**

The SIM_URRBMI1.0 consists of 28 measurement variables and 11 latent variables. It is a reliable, valid, and standard satisfaction measurement tool for URRBMI with good prediction ability for public satisfaction. In addition, the model provides an accurate evaluation of the perceived quality, which will greatly help with performance improvement diagnosis. The most critical aspects of satisfaction improvement are optimizing the scope and proportion of reimbursement as well as setting appropriate level of deductible and capitation of URRBMI.

**Supplementary Information:**

The online version contains supplementary material available at 10.1186/s12911-022-02002-5.

## Background

In 2021, the general office of the State Council issued the "14th five year plan" for national medical security, pointing out that it is necessary to accelerate the establishment of a medical security system covering the whole people and integrating urban and rural areas [1]. The Chinese government has been trying to establish a multi-level medical security system based on the social basic medical insurance schemes to protect the finances of individuals and families affected by illness and injury. China has integrated the new cooperative medical scheme (NCMS) and the urban resident-based basic medical insurance scheme (URBMI) into the urban and rural resident-based basic medical insurance scheme (URRBMI) [2, 3]. With the advancement of the social basic medical insurance reform, the evaluation model needs to be improved accordingly.

Voluntary enrollment is a principle of URRBMI, which means that it is particularly important to evaluate from the perspective of the insured [4]. The satisfaction and loyalty of participants are crucial to the effective implementation and sustainable development of the scheme. Moreover, analyzing the enrollee’s satisfaction with URRBMI and its influencing factors can help decision-making [2, 5, 6]. The American customer satisfaction index (ACSI) is one of the most widely used quantitative measurement scales of satisfaction. It shows the cause-and-effect relationship linking the causal variables of customer satisfaction with its consequent variables [7]. The ACSI has been widely used in the field of the economy, tourism, government agencies, and medical insurance [8–11]. Its advantage is that it provides standardized and comparable measurements of satisfaction and quantitative evaluations of the relationship between explanatory variables and satisfaction. Based on the ACSI, Peng et al. constructed a satisfaction index model for URRBMI with good reliability and validity. Furthermore, this model provides a standard measurement tool for URRBMI satisfaction, and guidance for satisfaction improvement strategies [8, 9]. However, Peng’s model only adopted a few indicators to assess perceived quality, with the overall quality assessment item. The insured’s perception of the quality of URRBMI involves multiple stakeholders: the policy formulation and service of the medical insurance management department, the medical service provided by the medical department, the insurance service provided by the community and the bank, etc. The lack of comprehensive and detailed aspects of URRBMI quality measurement makes it impossible to identify the important aspects of quality that need to be improved urgently and provide an accurate reference for policy optimization and service improvement [8, 9].

There is evidence that perceived quality may be the most worthwhile topic in the service area satisfaction research, and satisfaction is mainly determined by perceived quality [7, 12, 13]. The study aims to optimize the perceptive quality and revise and validate the SIM_URRBMI1.0, which could identify the main quality aspects that need to be improved and thereby provide a valid, reliable, and practical satisfaction measurement tool for URRBMI.

What is more, the credibility and effectiveness of patient-reported outcomes are increasingly valued, providing effective support for improving clinical care, promoting clinical decision-making, enhancing patients’ trust of medical staff and satisfaction with medical services, and providing a basis for the formulation of medical-related policies [14]. Although patient-reported outcomes are more and more widely used, their application is weak in the field of health policy formulation and improvement, and they are also easy to ignore and difficult to achieve. Therefore, we used self-reported satisfaction as the outcome effect, which directly reflects participants’ views on medical insurance.

The purpose of this study is to revise the SIM_URBMI to facilitate the evaluation of medical insurance policy and guide quality improvement. The first step is the determination of a model theoretical framework and the initial pool of measurement variables, and the assumption of the path relationship between the latent variables as well as the selection of measurement variables for the model. The second step is to test the reliability and validity of the model and measure the path relationship between latent variables.

## Methods

This section consists of three parts. First, we introduce the acquisition methods of research objects and samples, and in the following two subsections we describe the main process of model revision and validation.

### Participants and sampling

The survey was conducted in January 2018. It was school-based because it facilitated implementation and helped to achieve good participation rates. In addition, it might avoid the potential restrictions that the elderly were not clear about related policies or that transient young people were not at home. A three-stage randomized stratified cluster sampling method was used to ensure a representative sample. The first stage was to randomly select two districts/counties from the nine districts / counties of Changsha. The second stage was to randomly select four urban schools and four rural schools from 69 urban schools and 192 rural schools of the two selected districts/counties. Finally, for each selected school, one class was randomly selected for each grade, and all students from the selected classes were invited to participate. The questionnaire containing sociodemographic information on both the pupil and the most important decision maker on URRBMI in his/her family and assessed their satisfaction with URRBMI. Questionnaires were distributed to the subjects and collected the next day with the help of the teacher.

The sample was randomly divided into two parts, one (574, 30%) for the selection of measurement variables and the other (1335, 70%) for model evaluation. This study used a graded response model (GRM) to select the measurement variables. To ensure the accuracy of the estimated parameters, a sample size of at least 500 cases is needed [15]. For the partial least squares structural equation model (PLS-SEM), the sample size calculation formula using the inverse square root method is *n* > (2.486/*|β|*_min_)^2^. *|β|*_min_ refers to the absolute value of the statistically significant path coefficient with the minimum magnitude in the model [16]. Based on our pilot survey, *|β|*_min_ was 0.11, so the minimum sample size was calculated as 511. In addition, considering 20% lost or invalid responses and a multigroup analysis by urban and rural areas, the minimum sample size for partial least squares (PLS) was 1278. The sample size of this study met the requirements.

### Model construction

**Fig. 1:**
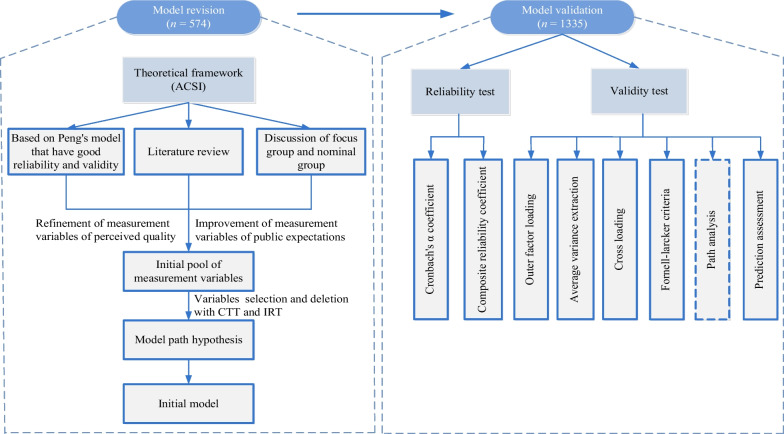
Model construction and validation flow chart

Model construction includes model revision and model validation (Fig. 1).

### Model revision

#### Phase 1

The programmed decision-making process, a method to simultaneously revise a scale using a focus group (the same research group used in Peng’s model, including two experts on epidemiology and health statistics, one health insurance administration, and four graduate students) and a nominal group (including pupils and their family members, local medical insurance management and service officers, experts in social medicine, health management, primary health care, and health statistics, and an epidemiologist), was used to construct the model. ACSI mainly studies American customers' evaluation of domestic product and service quality, and calculates the satisfaction index by constructing a model, which contains sets of causal relationships, and can indicate the relationship between the antecedents (customer expectations, perceived quality and perceived value) and the consequences (customer complaints and customer loyalty) of customer satisfaction [7]. Based on the theoretical framework of ACSI, insured residents can be regarded as "customers" and "customer satisfaction" refers to the evaluation of the implementation effect of URRBMI by insured residents from the perspective of URRBMI policy studied in this paper. Based on Peng’s model and studies on satisfaction measurement with ACSI [7, 8, 17–24], the focus group firstly explained the framework and definition, and stated the purpose. Then, the nominal group proposed item suggestions, which were discussed by the two groups and summarized into an initial item pool. The detailed process of model revision is as follows: (1) Firstly, from the perspective of the government departments, the word “customer” should be changed to “public”. (2) In addition, due to URRBMI’s authority, universality and high insurance participation rate, “customer loyalty” was difficult to be reflected in repeated insurance participation behavior but reflected in trust and support. Therefore, “customer loyalty” is changed to “public trust”, which is evaluated by two measurement variables of willingness to positively evaluate and willingness to recommend to others. (3) The latent variables of “perceived quality” were also refined. It was evaluated from multiple dimensions, mainly from the overall quality, information quality (policy publicity), service quality (staff service quality and attitude), product function quality (medical insurance policy), institution quality (quality of agency, designated medical institutions, designated pharmacies, etc.). The awareness degree of the insured on the medical insurance policy reflects the information openness degree or publicity intensity of government agencies. Therefore, the perceived information evaluation of the URRBMI was included in the perceived quality dimension, which is called “information quality”. Availability of relevant information (PQ2) and the convenience of obtaining relevant information (PQ3) are two measurement variables. URRBMI provide public service, and the “service quality” was defined as the attitude and professional level of URRBMI staff. Thus, the “service quality” corresponding to two observation variables respectively: service attitude of staff (PQ4) and clarity of interpretation of relevant policies (PQ5). Seven measurement variables were selected for policy quality: degree of meeting basic health insurance needs (PQ6), degree of meeting personalized health insurance needs (PQ7), payment level (PQ8), scope of reimbursement (PQ9), proportion of reimbursement (PQ10), deductible level (PQ11) and capitation (PQ12). “Quality of institutions” was used to assess the service quality of institutions in the process of enrollment, payment, compensation and receiving medical or pharmaceutical services. It was evaluated through five measurement variables, including service quality of community or village committees for enrollment (PQ13), quality of payment bank or online payment platform (PQ14), service quality of authorized medical institution (PQ15), service quality of reimbursement in medical institution (PQ16), service quality of authorized pharmacy (PQ17). In conclusion, the “perceived quality” was refined to five first-order latent variables with 17 measurement variables (PQ1-PQ17), namely, overall quality, information quality, service quality, policy quality, and institution quality (a summary of the literature on perceived quality is given in Additional file 1: Table S1) [7, 8, 17–28].

Public expectations refer to the expectation perception of insured residents on the protection level of URRBMI, including overall expectation (PE1), basic medical protection demand expectation (PE2), and special medical protection demand (PE3). The higher the expectation of URRBMI might lead to higher perceived quality, perceived value and satisfaction level of URRBMI. Therefore, the following hypotheses were proposed in this study:

##### Hypothesis 1A

Public expectations has a direct positive impact on perceived quality.

##### Hypothesis 1B

Public expectations has a direct positive impact on perceived value.

##### Hypothesis 1C

Public expectations has a direct positive impact on public satisfaction.

Perceived quality refers to the insured residents' perception of the quality of the information, services, policy guarantee and level of relevant institutions provided by URRBMI. Whether the quality of URRBMI can meet the needs of the insured will directly affect their perception of URRBMI’s value and satisfaction. Therefore, the following hypotheses were proposed in this study:

##### Hypothesis 2A

Perceived quality has a direct positive impact on perceived value.

##### Hypothesis 2B

Perceived quality has a direct positive impact on public satisfaction.

Perceived value refers to the comparison between the cost paid by insured residents and the service quality provided by URRBMI, including quality according to the payment (PV1) and payment according to the quality (PV2). If the insured think that the service quality of URRBMI is greater than its cost, it indicates the perceived value is high, and the degree of satisfaction is high. Therefore, the following hypothesis were proposed in this study:

##### Hypothesis 3

Perceived value has a direct positive impact on public satisfaction.

Public complaints refer to the negative reaction of insured residents to URRBMI, which is one of the results of dissatisfaction, including informal complaints about URRBMI (PC1) and formal complaints about URRBMI (PC2). On the contrary, public trust refers to the positive evaluation caused by the insured residents' satisfaction with URRBMI, including the willingness to make positive evaluation (PT1) and the willingness to recommend to others (PT2). Overall satisfaction (PS1), satisfaction compare to meet expectations (PS2) and proximity to the ideal basic medical insurance (PS3) were selected as measurement variables of public satisfaction. The more satisfied the insured is with URRBMI, the less they complain and have more trust in it. Therefore, the following hypotheses were proposed in this study:

##### Hypothesis 4

Public satisfaction has a direct positive impact on public trust.

##### Hypothesis 5

Public satisfaction has a direct negative impact on public complaints.

To sum up, an initial draft variable pool (29 variables) was generated in Table 1.

**Table 1:** The latent and measurement variables of the initial SIM_URRBMI

Latent variable	First-order latent variable	Measurement variables
Public Expectations		Overall expectations (PE1)
		Expectations of URRBMI to ensure basic medical needs (PE2)
		Expectations of URRBMI to ensure personalization of medical needs (PE3)
Perceived Quality	Overall quality	Overall evaluation of quality (PQ1)
	Information quality	Availability of related information (PQ2)
		The convenience of obtaining relevant information (PQ3)
	Service quality	Service attitude of staff (PQ4)
		Clarity of staff’s interpretation of relevant policies (PQ5)
	Policy quality	Degree of meeting basic health insurance needs (PQ6)
		Degree of meeting personalized health insurance needs (PQ7)
		Payment level (PQ8)
		Scope of reimbursement (PQ9)
		Reimbursement proportion (PQ10)
		Deductible level (PQ11)
		Capitation (PQ12)
	Quality of institutions	Service quality of community or village committees for enrollment (PQ13)
		Quality of payment bank or online payment platform (PQ14)
		Service quality of authorized medical institution (PQ15)
		Service quality of reimbursement in medical institution (PQ16)
		Service quality of authorized pharmacy (PQ17)
Perceived Value		Quality according to the payment (PV1)
		Payment according to the quality (PV2)
Public Satisfaction		Overall satisfaction (PS1)
		Satisfaction compare to expectations (PS2)
		Proximity to the ideal basic medical insurance (PS3)
Public Complaints		Complained informally about URRBMI (PC1)
		Complained formally about URRBMI (PC2)
Public Trust		Willingness to make positive evaluation (PT1)
		Willingness to recommend to others (PT2)

PE2 and PQ6 are about the expectation and perceived quality on URRBMI for basic medical needs(related to common diseases)

PE3 and PQ7 are about the expectation and perceived quality on URRBMI for some special medical demands (related to special disease outpatient service, such as chronic disease, serious illness, rare disease etc.

These patients usually need long periods of treatment with higher medical cost). PQ2 refers to how much relevant information is useable, while the PQ3 refers to how easy it is to access relevant information

#### Phase 2

Classic test theory (CTT), combined with item response theory (IRT), was applied for the measurement variable selection. If a measurement variable reached three or more criteria, it was considered for deletion. The selection methods and criteria are shown in Table 2. CTT is based on the true score model (true score plus error). The basic idea of IRT is to use a mathematical function to characterize the relationship between the test responses. In this study, *θ* refers to subject satisfaction with URRBMI. For the GRM, the discrimination parameter (*a*), threshold parameter (*b*), and item information function *(IIF*) were used to evaluate the quality of measurement variables. In addition, the test information function (*TIF*) is a linear cumulative measurement of each item *IIF*—that is, the test information function on the *θ* value is equal to the sum of its *IIFs*. The quality of the model is good when the total of the information function is greater than 25, while the quality is considered poor when the information function is less than 16 [9, 29]. Since the initial draft of the SIM_URRBMI contains a total of 29 measurement variables, the average information function amount of each measurement variable required is ($\overline{I}$) ≥ 0. 55 (16 / 29). The deletion criteria based on IRT are as follows: (1) *a* is less than 0.3; (2) *b* is out of range of (‒4, 4); and (3) $\overline{I}$ is less than 0.55.

**Table 2:** Item selection methods and potential deletion criteria based on CTT and IRT

Methods	Criteria	Test aspects
Floor effect	20% or more individuals choose the lowest score value	Sensitivity
Ceiling effect	20% or more individuals choose the highest score value	Sensitivity
*t*-test	The measurement variables with no statistically significant difference (α = 0.05) between 27% of the individual groups with the highest and lowest satisfaction scores	Sensitivity or discrimination
Cronbach’s *α* coefficient	There is a large increase in the Cronbach’s *α* after the removal of a measurement variable	Internal consistency
Item-dimension Correlation	The Pearson correlation coefficient of each measurement variable score and its corresponding latent variable score is less than 0.6	Sensitivity or representation
Item-total Correlation	The Pearson correlation coefficient of each measurement variable score and the model total score is less than 0.4	Representation
Stepwise regression	Using the total score of each latent variable as the dependent variable, the stepwise regression analysis is performed with candidate measurement variables as independent variables (*sle* = 0. 05, *sls* = 0. 10)	Importance
Exploratory Factor analysis	Measurement variables with a factor load less than 0.4	Representation
*a*	Less than 0.3	Discrimination
*b*	Out of range of (‒4, 4)	Degree of under standability
$\overline{I}$	$\overline{I}$ Less than 0. 55	Estimated accuracy

### Model validation

PLS-SEM are more suitable for skew distribution data and higher-order constructs (HOC) [30, 31]. The satisfaction data are often positively skewed, and HOC was included in our model. We selected the PLS-SEM for model validation. The model validation includes two parts. The reliability and validity tests were used to evaluate the measurement model. The value of Cronbach’s *α* coefficient and the composite reliability coefficient were greater than 0.7, which is considered to indicate good reliability, and higher values indicate greater homogeneity. Outer factor loading (≥ 0.7) and average variance extraction (*AVE*) (≥ 0.5) were used to assess the convergence validity, while cross-loading and the Fornell‒Larcker criteria were used to assess discriminant validity. Good discriminant validity means that the outer loading of a measurement variable on its corresponding latent variable is greater than its cross-loadings on other latent variables, or the square root of the *AVE* of each latent variable is greater than its linear correlation coefficients with any other latent variables. Path analysis of the latent variables and model prediction were used to evaluate the structural model [30]. The relationship between latent variables was evaluated by estimating the direct effect, indirect effect, and total effect. The model prediction assessment indicators were the adjusted coefficient of determination (*R*^2^_adj_) (in general, *R*^2^_adj_ values of 0.25, 0.50, and 0.75 for target constructs are considered weak, medium, and substantial, respectively), the effect size (*f* ^2^) (results of 0.02, 0.15, and 0.35 are interpreted as small, medium, and large effect sizes, respectively), and the predictive relevance (*Q*^2^) (the path model has predictive relevance for a selected endogenous construct if the *Q*^2^ value is above 0).

### Statistical analysis

A second-order partial least squares structural equation model (PLS-SEM) was used to test the model’s reliability and validity, and PLS-SEM multigroup analysis (PLS-MGA) was used to explore the heterogeneity between rural and urban participants. A two-tailed *P* ≤ 0.05 was considered statistically significant.

The descriptive statistics and CTT analyses were conducted using SPSS 18.0. The IRT analysis was performed using Mplus7.0. PLS-SEM and PLS-MGA analyses were conducted with SmartPLS3.0.

## Results

### Samples

A total of 1909 participants who had insurance for their children were included in the primary data analysis. Table 3 shows the sociodemographic information of pupils and main decision makers. For pupils, the mean age was 9.26 ± 1.75, and the proportions of male (945, 49.50%) and female students (949, 49.71%) were similar. For the main decision makers, the mean age was 37.13 ± 5.90 years; most decision makers were parents of pupils (1845, 96.95%), especially mothers (1196, 62.65%). There were no significant differences in sociodemographic information of the pupils and the main decision makers for their URRBMI between the model construction group and model evaluation group, suggesting that the two groups were homogenous. The measurement scores and comparative analysis between the two groups are shown in Additional file 1: Table S2.

**Table 3:** Sociodemographic information of the pupils and the main decision makers for their URRBMI (n = 1909)

Variables	Mean ± SD / *n* (%)
*Pupils*	
Age, Mean ± SD	9.26 ± 1.75
*Area, n (%)*	
urban	899 (46.57%)
rural	1020 (53.43%)
*Sex, n (%)*	
Male	945 (49.50%)
Female	949 (49.71%)
Missing data	15 (0.79%)
*Health condition, n (%)*	
Very good	1051 (55.06%)
Good	751 (39.34%)
General	91 (4.77%)
Missing data	16 (0.84%)
*Main decision makers*	
Age, Mean ± SD	37.13 ± 5.90
*Relationship with pupils, n (%)*	
Father	649 (34.00%)
Mother	1196 (62.65%)
Other	58 (3.04)
Missing data	6 (0.31%)
*Sex, n (%)*	
Male	674 (35.31%)
Female	1229 (64.38%)
Missing data	6 (0.31%)
*Marital status, n (%)*	
Married	1827 (96.70%)
Divorced	56 (2.93%)
Never married	4 (0.21%)
Other	5 (0.26%)
Missing data	17 (0.89%)
*Education, n (%)*	
Junior high school and below	535 (28.03%)
High school or technical secondary school	822 (43.06%)
Junior college	281 (14.72%)
Bachelor’s	210 (11.00%)
Master’s or above	25 (1.31%)
Missing data	36 (1.89%)

*SD* standard Deviation

### Model construction

Additional file 1: Table S3 provided GRM estimations of the discrimination (*a*) and threshold parameters (*b*1‒*b*9) as well as their standard errors (SE). Table 4 shows the results of measurement variable selection. Items were marked when they meet the deletion criteria. Using these methods, five variables (PQ1, PQ14, PE2, PT1, and PT2) were marked with the ceiling effect, one variable (PS3) was marked with Cronbach’s *α*, one variable (PC1) was marked with *a*, nine variables (PQ9‒PQ12, PQ16, PV1, PV2, PS1, and PS2) were marked with *b*, and four variables (PQ1, PQ2, PC1, and PC2) were marked with $\overline{I}$.

**Table 4:** The results of measurement variable selection with the methods of CTT and IRT

Variables	CTT								IRT			Selected
	Floor effect	Ceiling effect	*t-test*	Item-dimension Correlation	Item-total Correlation	Cronbach’s *α *coefficient	Stepwise regression	Factor analysis	*a*	*b*	$\overline{I}$	
PE1												0
PE2		** × **										1
PE3												0
PQ1		** × **									** × **	2
PQ2											** × **	1
PQ3												0
PQ4												0
PQ5												0
PQ6												0
PQ7												0
PQ8												0
PQ9										** × **		1
PQ10										** × **		1
PQ11										** × **		1
PQ12										** × **		1
PQ13												0
PQ14		** × **										1
PQ15												0
PQ16										** × **		1
PQ17												0
PV1										** × **		1
PV2										** × **		1
PS1										** × **		1
PS2										** × **		1
PS3						** × **						1
PC1	–	–							** × **		** × **	2
PC2	–	–									** × **	1
PT1		** × **										1
PT2		** × **										1

** × **Suggested deletion, *CTT* classic test theory, *IRT* item response theory, *PE* public expectations, *PQ* perceived quality, *PV* perceived value, *PS* public satisfaction, *PC* public complaints, *PT* public trust

**Fig. 2:**
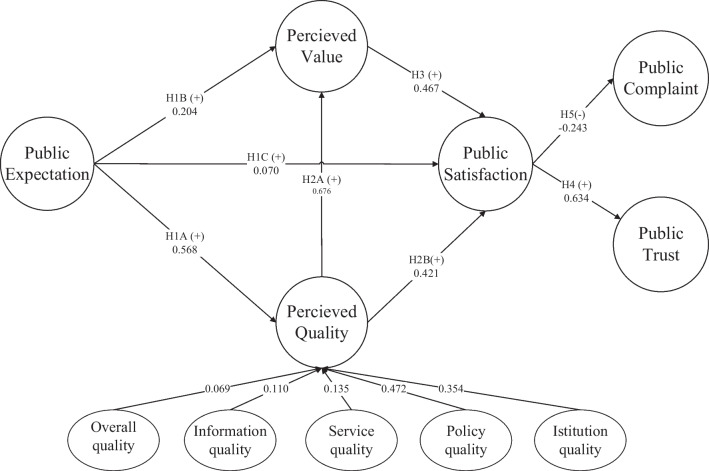
The SIM_URRBMI1.0 and direct path [39]

Overall, no item reached deletion criteria (being marked three or more times). However, it can be seen that the $\overline{I}$ of PC2 was 0.005, suggesting that its contribution to the model was near 0, and its discrimination was also low (*a* is 0.351). In addition, given Chinese social and cultural background, most enrollees will not formally complain even if they were dissatisfied. Thus, the PC2 was deleted, and the remaining 28 variables were used to construct the SIM_URRBMI1.0 (Fig. 2).

### Model validation

#### Reliability

The Cronbach’s *α* coefficient for each of the latent variables ranged from 0.772 to 0.956, and the composite reliability coefficients ranged from 0.897 to 0.960, both of which are greater than 0.7, providing evidence that the SIM_URRBMI1.0 is reliable (Table 5).

**Table 5:** The result of a reliability test of the SIM_URRBMI1.0

Latent variables	Cronbach's α coefficient	Composite reliability coefficient
PE	0.918	0.948
PQ	0.956	0.960
PV	0.895	0.950
PS	0.867	0.919
PC	−	−
PT	0.772	0.897
PQ_overall	–	–
PQ_information	0.796	0.907
PQ_service	0.845	0.928
PQ_policy	0.933	0.946
PQ_institution	0.926	0.944

*PE* public expectations, *PQ* perceived quality, *PV* perceived value, *PS* public satisfaction, *PC* public complaints, *PT* public trust

#### Validity

The results of the convergent and discriminant validity of the SIM_URRBMI1.0 are summarized in Tables 6, 7 and 8. The *AVEs* of all latent variables were greater than 0.5, and the outer factor loadings of all measurement variables ranged from 0.726 to 1.000, except the second-order external factor loadings for PQ1, PQ2, and PQ3 (0.692, 0.595, and 0.684, respectively), which were slightly below the critical value of 0.708, showing good convergence validity (Table 6). All outer loadings of the measurement variables on their corresponding latent variables were greater than their cross-loadings on other latent variables (Table 7). The square roots of *AVEs* of each latent variable was greater than the linear correlation between the latent variable and other structures, except for the perceived quality variable, which has a slightly higher correlation with perceived value and public satisfaction (Table 8). The results indicate good discriminant validity. All but one of the paths were statistically significant and positive. The effect of public satisfaction on public complaints was negative, which is in line with the model theory framework. Table 9 and Additional file 1: Table S4 show the results of the direct effects and the collinearity assessment of the SIM_URRBMI1.0. Furthermore, the greatest effect of the five first-order latent variables on perceived quality was medical insurance policy quality (0.472), followed by institutional quality (0.354) (Table 9). All the value of variance inflation factor (*VIF*) were clearly below the threshold of 5. Therefore, there was no collinearity between the predictor constructs in the structural model. The two paths with the greatest direct effect were perceived quality on perceived value (0.676) and public satisfaction on public trust (0.634). Additional file 1: Table S5 indicates the indirect effects of the SIM_URRBMI1.0. The total effect of the 14 paths of the SIM_URRBMI1.0 were all statistically significant, showing that the model was appropriate. Among the three explanatory variables of public satisfaction, the greatest total effect came from perceived quality (0.737).

**Table 6:** The result of convergence validity test for the SIM_URRBMI1.0

Latent variables	Measurement variable	Outer factor loading	Outer factor loading (second order)	*AVE*
PE	PE1	0.921	–	
	PE2	0.943	–	
	PE3	0.916	–	
PQ	–	–	–	0.590
PQ_overall	PQ1	1.000	0.692	1.000
PQ_information	PQ2	0.898	0.595	0.830
	PQ3	0.924	0.684	
PQ_service	PQ4	0.930	0.756	0.866
	PQ5	0.931	0.761	
PQ_policy	PQ6	0.788	0.757	0.716
	PQ7	0.783	0.731	
	PQ8	0.796	0.750	
	PQ9	0.901	0.833	
	PQ10	0.903	0.836	
	PQ11	0.884	0.832	
	PQ12	0.859	0.801	
PQ_institution	PQ13	0.856	0.783	0.759
	PQ14	0.836	0.726	
	PQ15	0.912	0.839	
	PQ16	0.920	0.844	
	PQ17	0.869	0.794	
PV	PV1	0.951	–	0.905
	PV2	0.952	–	
PS	PS1	0.925	–	0.791
	PS2	0.928	–	
	PS3	0.811	–	
PC	PC1	1.000	–	1.000
PT	PT1	0.887	–	0.813
	PT2	0.916	–	

*AVE* average variance extraction, *PE* public expectations, *PQ* perceived quality, *PV* perceived value, *PS* public satisfaction, *PC* public complaints, *PT* public trust

**Table 7:** The results of outer factor loadings and cross-loadings of the SIM_URRBMI1.0

Variables	PQ_overall	PQ_information	PQ_service	PQ_policy	PQ_institution	PQ (second order)	PE	PV	PS	PC	PT
PQ1	**1.000**	0.499	0.582	0.586	0.597	**0.692**	0.427	0.561	0.584	‒0.224	0.474
PQ2	0.428	**0.898**	0.496	0.515	0.439	**0.595**	0.287	0.444	0.460	‒0.118	0.305
PQ3	0.479	**0.924**	0.619	0.588	0.529	**0.684**	0.333	0.482	0.515	‒0.132	0.375
PQ4	0.577	0.544	**0.930**	0.595	0.703	**0.756**	0.440	0.565	0.597	‒0.216	0.467
PQ5	0.507	0.603	**0.931**	0.645	0.645	**0.761**	0.434	0.568	0.604	‒0.204	0.416
PQ6	0.533	0.546	0.610	**0.788**	0.584	**0.757**	0.448	0.596	0.624	‒0.196	0.439
PQ7	0.442	0.537	0.566	**0.783**	0.556	**0.731**	0.389	0.540	0.591	‒0.190	0.392
PQ8	0.505	0.478	0.514	**0.796**	0.612	**0.750**	0.455	0.671	0.633	‒0.267	0.524
PQ9	0.521	0.516	0.578	**0.901**	0.666	**0.833**	0.394	0.674	0.698	‒0.260	0.476
PQ10	0.512	0.500	0.565	**0.903**	0.681	**0.836**	0.421	0.680	0.708	‒0.238	0.520
PQ11	0.494	0.522	0.570	**0.884**	0.690	**0.832**	0.409	0.681	0.684	‒0.262	0.504
PQ12	0.463	0.502	0.544	**0.859**	0.658	**0.801**	0.366	0.652	0.672	‒0.280	0.481
PQ13	0.490	0.456	0.675	0.644	**0.856**	**0.783**	0.493	0.590	0.650	‒0.213	0.517
PQ14	0.507	0.418	0.583	0.573	**0.836**	**0.726**	0.478	0.565	0.597	‒0.193	0.503
PQ15	0.546	0.505	0.653	0.706	**0.912**	**0.839**	0.521	0.678	0.735	‒0.237	0.568
PQ16	0.553	0.507	0.663	0.706	**0.920**	**0.844**	0.534	0.693	0.747	‒0.255	0.575
PQ17	0.526	0.458	0.608	0.671	**0.869**	**0.794**	0.535	0.629	0.671	‒0.207	0.539
PE1	0.397	0.332	0.449	0.448	0.559	0.534	**0.921**	0.537	0.546	‒0.083	0.486
PE2	0.391	0.297	0.444	0.437	0.542	0.517	**0.943**	0.547	0.541	‒0.074	0.491
PE3	0.400	0.322	0.411	0.468	0.520	0.524	**0.916**	0.544	0.531	‒0.052	0.454
PV1	0.540	0.484	0.577	0.726	0.685	0.755	0.556	**0.951**	0.795	‒0.293	0.601
PV2	0.528	0.485	0.581	0.721	0.684	0.752	0.557	**0.952**	0.807	‒0.264	0.589
PS1	0.581	0.493	0.629	0.741	0.754	0.797	0.559	0.831	**0.925**	‒0.297	0.598
PS2	0.544	0.513	0.616	0.743	0.729	0.787	0.528	0.791	**0.928**	‒0.276	0.563
PS3	0.419	0.420	0.462	0.583	0.571	0.615	0.461	0.604	**0.811**	‒0.212	0.526
PC1	‒0.275	‒0.169	‒0.277	‒0.351	‒0.310	‒0.351	‒0.093	‒0.359	‒0.364	**1.000**	‒0.251
PT1	0.389	0.298	0.390	0.472	0.520	0.520	0.456	0.509	0.530	‒0.142	**0.887**
PT2	0.462	0.376	0.461	0.542	0.587	0.600	0.472	0.613	0.607	‒0.223	**0.916**

The bold numbers in the table are the outer factor loadings of the measurement variables on its corresponding latent variable, and the rest are the cross-loadings

*PQ* perceived quality, *PE* public expectations, *PV* perceived value, *PS* public satisfaction, *PC* public complaints, *PT* public trust

**Table 8:** The results of the Fornell‒Larcker standard for the SIM_URRBMI1.0

	PQ	PQ_ overall	PQ_ information	PQ_ service	PQ_ policy	PQ_ institution	PE	PV	PS	PC	PT
PQ	**0.768**										
PQ_overall	0.692	**1.000**									
PQ_Information	0.705	0.499	**0.911**								
PQ_service	0.815	0.582	0.616	**0.931**							
PQ_policy	0.937	0.586	0.608	0.666	**0.846**						
PQ_institution	0.908	0.597	0.534	0.724	0.753	**0.879**					
PE	0.567	0.427	0.342	0.470	0.486	0.583	**0.927**				
PV	0.792	0.561	0.510	0.609	0.760	0.720	0.585	**0.951**			
PS	0.830	0.584	0.536	0.646	0.780	0.776	0.582	0.842	**0.890**		
PC	‒0.286	‒0.224	‒0.138	‒0.226	‒0.286	‒0.252	‒0.076	‒0.293	‒0.297	**1.000**	
PT	0.623	0.474	0.376	0.475	0.565	0.616	0.515	0.625	0.633	‒0.205	**0.902**

The bold numbers in the table are the square roots of AVE for each latent variable

*PQ* perceived quality, *PE* public expectations, *PV* perceived value, *PS* public satisfaction, *PC* public complaints, *PT* public trust

**Table 9:** The results of the direct effects analysis for the SIM_URRBMI1.0

Direct path	Coefficient	SE	*T*	*P*	95% CI	
					Lower	Upper
PE → PQ	0.568	–	–	–	–	–
PE → PV	0.204	0.027	7.372	< 0.001	0.145	0.253
PE → PS	0.070	0.021	3.362	0.001	0.034	0.119
PQ → PV	0.676	0.021	31.964	< 0.001	0.639	0.720
PQ → PS	0.421	0.028	14.960	< 0.001	0.361	0.474
PV → PS	0.467	0.032	14.698	< 0.001	0.404	0.528
PS → PC	‒0.243	0.020	11.847	< 0.001	‒0.280	‒0.202
PS → PT	0.634	0.020	31.305	< 0.001	0.596	0.674
PE → PQ_overall	0.428	0.027	15.651	< 0.001	0.373	0.477
PE → PQ_information	0.344	0.026	12.981	< 0.001	0.290	0.393
PE → PQ_service	0.471	0.026	18.247	< 0.001	0.413	0.518
PE → PQ_policy	0.487	0.025	19.415	< 0.001	0.437	0.534
PE → PQ_institution	0.584	0.023	25.431	< 0.001	0.534	0.624
PQ_overall → PQ	0.069	0.002	36.692	< 0.001	0.066	0.073
PQ_information → PQ	0.110	0.003	31.786	< 0.001	0.103	0.117
PQ_service → PQ	0.135	0.003	47.898	< 0.001	0.130	0.142
PQ_policy → PQ	0.472	0.006	76.267	< 0.001	0.462	0.485
PQ_institution → PQ	0.354	0.005	64.894	< 0.001	0.343	0.364

*SE* standard error, *PE* public expectations, *PQ* perceived quality, *PV* perceived value, *PS* public satisfaction, *PC* public complaints, *PT* public trust

The adjusted coefficient of determination (*R*^2^_adj_) of each endogenous latent variable ranged from 0.088 to 1.000. The* R*^2^_adj_ values were 0.783 and 0.400 for public satisfaction and public trust, respectively, indicating a strong and moderate prediction. The effect size (*f* ^2^) ranged from 0.014 to 0.903. The *f* ^2^ values for perceived quality on predicted perceived value, the perceived value on predicted public satisfaction, and public satisfaction on predicted public trust were 0.903, 0.351, and 0.667, respectively. All the values were greater than 0.35, indicating a strong prediction effect. The predictive relevance (*Q*^2^) of endogenous latent variables were all greater than 0, ranging from 0.105 to 0.585, showing a certain predictive relevance for all five endogenous latent variables (Table 10).

**Table 10:** The predictive ability of the SIM_URRBMI1.0

Indicators	PE	PQ	PV	PS	PC	PT
R^2^_adj_	–	1.000	0.654	0.783	0.088	0.400
The predictive correlation (*Q*^2^)	–	0.550	0.567	0.585	0.105	0.311
The effect scale (*f *^*2*^)						
PE	**–**	–	0.080	0.014	**–**	–
PQ	–	**–**	0.903	0.290	–	–
PV	**–**	–	**–**	0.351	–	–
PS	–	**–**	**–**	**–**	0.097	0.667

*PE* public expectations, *PQ* perceived quality, *PV* perceived value, *PS* public satisfaction, *PC* public complaints, *PT* public trust

## Discussion

To meet participants’ growing need for a better life, there is an urgent need to understand participants’ satisfaction with the basic medical insurance system and to diagnose the main aspects that need to be improved [5]. Based on the ACSI, this study constructed a valid and reliable satisfaction measurement tool suitable for URRBMI in China. It can provide a standard, comparable, and dynamic evaluation of the performance of URRBMI. Based on the PLS-SEM, SIM_URRBMI1.0 has the following characteristics: (1) Based on the regionalization of URRBMI policy and the dynamic promotion of medical insurance reform in China, this model makes it possible to compare the reform performance in different regions and periods, since it can provide standard evaluation and ensure good comparability. (2) By constructing second-order latent variables, the measurement of perceived quality is refined. This improvement provides a measurement basis for quality diagnosis and quality improvement feedback.

The quality of medical insurance determines the satisfaction of the insured to a large extent. The original SIM_URRBMI developed by Peng et al. provides a standard measure of satisfaction, but due to the lack of detailed quality measurement variables, it is impossible to diagnose the aspects of poor quality in detail. This study constructed the perceived quality variable as a second-order latent variable based on the literature review and expert consultation. The second-order SEM can not only reduce the number of structural model path relationships but also make the model paths more concise and easier to understand. Most importantly, the quality perception of URRBMI participants can be measured more accurately by the second-order SEM. This characteristic will help identify the aspects of URRBMI that are in urgent need of improvement [31]. The research results of detailed measurement of quality show that URRBMI could be improved from the following four aspects: (1) The most concerned and important core part of the policy is that the government needs to gradually expand the scope of reimbursement, increase the proportion of reimbursement, optimize the deductible and the capitation, so as to improve the participants’ satisfaction. (2) Optimize the management of designated medical institutions and improve medical quality and medical insurance services. (3) Government departments should adhere to refined management and high-quality services to provide more intimate and warm services for the people, such as online insurance enrollment and electronic medical insurance settlement, etc. (4) Strengthen the publicity of policies and health education, so that the participants can better understand the medical insurance policies and procedures, and know how to use medical insurance services. The results of the predictive ability evaluation showed that the* R*^2^_adj_ of public satisfaction was 0.783, which is larger than the *R*^2^ of public satisfaction in the original version of SIM_URRBMI constructed by Peng et al. (0.63). So, the construction of PQ as a second-order latent variable in this study increased the predictive power of public satisfaction. This also verifies our hypothesis that the quality-refined model can measure public satisfaction more accurately.

As one of the key stakeholders, patients are more qualified to have a say in the improvement of healthcare decisions and healthcare satisfaction. We take patient-reported satisfaction as the research content, which directly reflects the views of patients on the URRBMI policy and process of enrollment, payment, medical and pharmaceutical services, reimbursement. At present, few studies use patient-reported satisfaction results to help decision-making, and one of the reasons is that there is no appropriate measurement tool [14, 32]. Therefore, this study can effectively solve the problem of insufficient measurement tools for patient-reporting satisfaction in the current reform of URRBMI.

The measurement variables selection is a key step in model construction. CTT and IRT complement each other, so combining the methods in measurement variable selection will provide more reliable choices for model construction. The measurement variable PC2 (formal complaint) had poor performance, which is consistent with Chinese culture in that the public seldom chooses this way to express their dissatisfaction with public services. Deletion of PC2 suggested that this study is appropriate for measurement variable selection methods [19].

Compared with covariance-based SEM (CB-SEM), PLS-SEM is more suitable for exploration, but not for validation analysis. It makes more liberal assumptions about the distribution of data, and can explain the variance of potential variables to the greatest extent on the basis of minimizing the error term, so as to obtain the maximum model interpretation and prediction ability [10, 31, 33, 34]. In addition, when there is a higher-order construct (HOC), the parameter estimation of PLS-SEM is more accurate [31]. Since this study focuses on exploring the relationship between satisfaction and its related factors and the satisfaction data are skewed distribution, PLS-SEM is more suitable for this study.

The Cronbach’s *α* coefficients and composite reliability coefficients for all latent variables were greater than 0.7, suggesting good internal consistency except that the AVE of perceived quality is slightly less than its linear correlation coefficient with perceived value and public satisfaction, it may be related to the fact that the second-order factor load of PQ1-PQ3 in perceived quality is slightly less than 0.7. All the other results indicate that the model has good convergent validity and discriminant validity. Among all paths, the highest total effect is the impact of perceived quality on public satisfaction (0.737), indicating that PQ is the most important factor affecting public satisfaction, which is consistent with other researches [35, 36].

From the results of the path coefficients between perceived quality and its first-order latent variables, the quality of URRBMI policy had the greatest impact on perceived quality with a total effect of 0.472, suggesting that basic medical insurance policy also had the greatest impact on public satisfaction. Second, PQ9 (scope of reimbursement), PQ10 (reimbursement proportion), PQ11 (deductible level), and PQ12 (capitation) had larger second-order factor loadings on PQ. Moreover, PQ9-PQ12 had larger discrimination parameters (2.4–2.8) from the *IRT* and *IIF* results. According to Qin, the discrimination parameter reflects the sensitivity, which can reflect whether the relevant issues are of concern to each respondent, while the *IIF* result reflects that the insured residents are more concerned about the policies related to reimbursement [37]. Therefore, the reimbursement policy for URRBMI has the greatest impact on the satisfaction of insured residents, suggesting that improving the quality, especially the quality of medical insurance reimbursement policy, is crucial for improving the satisfaction of URRBMI.

In terms of the representativeness of the sample, first of all, although the policy strategies of URRBMI are different across the country, the framework of China’s basic medical insurance scheme is basically the same. Secondly, subtle differences of policies in different regions were considered in the study design, and the observation variables were set according to the general aspects of URRBMI. Finally, a remarkable feature of the model based on ACSI is that they are comparable among different regions [38]. Therefore, although the model validation was based on data of Changsha, theoretically it can be applied to the whole country. We will continue to track the feedback in the application and make improvements.

This study also has some limitations. In China's urban areas, employees are covered by the Basic Medical Insurance for Urban Employees (UEBMI). The insured residents of URRBMI are mainly the elderly, unemployed, teenagers and children without UEBMI. Thus, this study chose the primary school where one of the main participants of URRBMI gathered as the research site. The main family decision-makers of primary school students who complete the questionnaire are all have the experience of enrollment and payment and 36% of them had been ill or incurred non-deductible medical expenses in past one year which were considered likely to have reimbursement experience. In addition, they often responsible for the health and life of children and the elderly in the family, and they are most likely to understand the insurance policy. They could integrate all family members' feelings about URRBMI and conduct satisfaction assessment [39]. Considering that the purpose of this study is to revise the satisfaction measurement tool, rather than the survey of population satisfaction level, this study did not include all kinds of insured subjects of URRBMI. However, the research results should be extrapolated carefully, and future studies will try to expand the sample range for further evaluation. In addition, this study is a cross-sectional study without longitudinal data, so it is impossible to estimate the trend of URRBMI satisfaction in the sample area.

## Conclusions

The SIM_URRBMI1.0 consists of 28 measurement variables and 11 latent variables. It is a reliable, valid, and standard satisfaction measurement tool for URRBMI with good prediction ability for public satisfaction and public trust. In addition, the model provides an accurate evaluation of the perceived quality, which will greatly help with performance improvement diagnosis. The most critical aspects of satisfaction improvement are optimizing the scope and proportion of reimbursement as well as setting appropriate level of deductible and capitation of URRBMI.

## Supplementary Information

**Additional file 1:** Supplementary Material. **Table S1.** Summary of literature on the connotation of perceived quality. **Table S2.** The measurement scores and comparative analysis between two groups of the initial draft of SIM_URRBMI. **Table S3.** Item parameter estimation from the GRM. **Table S4.** The result of the collinearity assessment in the revised SIM_URRBMI1.0. **Table S5.** The indirect effects in the revised SIM_URRBMI1.0.

## Data Availability

The datasets generated and/or analyzed during the current study are not publicly available due to the confidential nature of the study interviews, but are available from the corresponding author on reasonable request.

## Abbreviations

ACSI: American customer satisfaction index
AVE: Average variance extraction
CTT: Classic test theory
*f*^2^: Effect size
GRM: Graded response model
HOC: Higher-order constructs
IIF: Item information function
IRT: Item response theory
NCMS: New cooperative medical scheme
PC: Public complaints
PE: Public expectations
PLS: Partial least squares
PLS-SEM: Partial least squares structural equation model
PQ: Perceived quality
PS: Public satisfaction
PT: Public trust
PV: Perceived value
*Q*^2^: Predictive relevance
R^2^_adj_: Adjusted coefficient of determination
SD: Standard deviation
SIM_URRBMI: Satisfaction index model for urban and rural resident-based basic medical insurance scheme
TIF: Test information function
UEBMI: Basic medical insurance for urban employees
URBMI: Urban resident-based basic medical insurance scheme
URRBMI: Urban and rural resident-based basic medical insurance scheme
