# Supplementary material for: Construction and validation of a revised satisfaction index model for the Chinese urban and rural resident-based basic medical insurance scheme
Source: BMC Med Inform Decis Mak. 2022 Oct 3;22:259. doi: 10.1186/s12911-022-02002-5 (PMC9531354; doi:10.1186/s12911-022-02002-5)
Supplement: Supplementary file 1 — Additional file 1: Supplementary Material. Table S1. Summary of literature on the connotation of perceived quality. Table S2. The measurement scores and comparative analysis between two groups of the initial draft of SIM_URRBMI. Table S3. Item parameter estimation from the GRM. Table S4. The result of the collinearity assessment in the revised SIM_URRBMI1.0. Table S5. The indirect effects in the revised SIM_URRBMI1.0. [file 12911_2022_2002_MOESM1_ESM.docx]

**Supplementary Table1 Summary of literature on the connotation of perceived quality**

| **Author** | **Latent variables** | **Measurement variables** |
| --- | --- | --- |
| Liai, P; Masoud Y [8,20] | Overall quality、Service quality、Policy quality | PQ1、PQ4、PQ5、PQ6、PQ7、PQ13、PQ16 |
| Zheng, G; Phonthanukitithaworn C [17,21] | Information quality、Service quality | PQ2、PQ3、PQ4、PQ5 |
| Lang, X [25] | Information quality | PQ2、PQ3 |
| Lijuan, J [26] | Quality of institutions | PQ13、PQ16 |
| Juanjuan, J; Qiuxian H [22,27] | Quality of institutions | PQ15、PQ16 |
| Yuan, Z; W.J. Deng a [23,28] | Information quality、Service quality、Quality of institutions | PQ2、PQ4、PQ14、PQ15、PQ16 |
| Xinyu, Wang; Masoud Y [18,20] | Policy quality | PQ6、PQ7、PQ8、PQ9、PQ10、PQ11、PQ12 |
| Tao, X; Hsu, SH [19,24] | Information quality、Service quality、Quality of institutions | PQ2、PQ3、PQ4、PQ5、PQ14、PQ15、PQ16、PQ17 |

**Supplementary Table2 The measurement scores and comparative analysis between two groups of the initial draft of SIM_URRBMI**

| **Variables** | **Measurement variable selection group（*n*=574）** | | | |  | **Model evaluation group （*n*=1335）** | | | |  | **All（*n*=1909）** | | | | **Statistics** | ***P*** |
| --- | --- | --- | --- | --- | --- | --- | --- | --- | --- | --- | --- | --- | --- | --- | --- | --- |
|  | **$\overline{X}$** | **$S$** | **$X_{min}$** | **$X_{\max}$** |  | **$\overline{X}$** | **$S$** | **$X_{min}$** | **$X_{\max}$** |  | **$\overline{X}$** | **$S$** | **$X_{min}$** | **$X_{\max}$** |  |  |
| PQ1 | 6.96 | 2.35 | 1.00 | 10.00 |  | 7.11 | 2.36 | 1.00 | 10.00 |  | 7.07 | 2.36 | 1.00 | 10.00 | 1.248 | 0.212 |
| PQ2 | 5.26 | 2.72 | 1.00 | 10.00 |  | 5.32 | 2.78 | 1.00 | 10.00 |  | 5.30 | 2.76 | 1.00 | 10.00 | 0.479 | 0.632 |
| PQ3 | 5.48 | 2.58 | 1.00 | 10.00 |  | 5.51 | 2.65 | 1.00 | 10.00 |  | 5.50 | 2.63 | 1.00 | 10.00 | 0.221 | 0.825 |
| PQ4 | 6.87 | 2.27 | 1.00 | 10.00 |  | 6.74 | 2.33 | 1.00 | 10.00 |  | 6.78 | 2.31 | 1.00 | 10.00 | 1.171 | 0.242 |
| PQ5 | 6.27 | 2.45 | 1.00 | 10.00 |  | 6.13 | 2.53 | 1.00 | 10.00 |  | 6.17 | 2.51 | 1.00 | 10.00 | 1.126 | 0.260 |
| PQ6 | 5.91 | 2.27 | 1.00 | 10.00 |  | 5.86 | 2.33 | 1.00 | 10.00 |  | 5.87 | 2.31 | 1.00 | 10.00 | 0.458 | 0.647 |
| PQ7 | 5.44 | 2.36 | 1.00 | 10.00 |  | 5.44 | 2.41 | 1.00 | 10.00 |  | 5.44 | 2.40 | 1.00 | 10.00 | 0.037 | 0.970 |
| PQ8 | 5.95 | 2.49 | 1.00 | 10.00 |  | 5.91 | 2.51 | 1.00 | 10.00 |  | 5.92 | 2.50 | 1.00 | 10.00 | 0.264 | 0.792 |
| PQ9 | 5.41 | 2.44 | 1.00 | 10.00 |  | 5.47 | 2.48 | 1.00 | 10.00 |  | 5.45 | 2.47 | 1.00 | 10.00 | 0.497 | 0.619 |
| PQ10 | 5.26 | 2.46 | 1.00 | 10.00 |  | 5.40 | 2.45 | 1.00 | 10.00 |  | 5.36 | 2.45 | 1.00 | 10.00 | 1.198 | 0.231 |
| PQ11 | 5.27 | 2.45 | 1.00 | 10.00 |  | 5.38 | 2.41 | 1.00 | 10.00 |  | 5.35 | 2.42 | 1.00 | 10.00 | 0.964 | 0.335 |
| PQ12 | 5.13 | 2.35 | 1.00 | 10.00 |  | 5.25 | 2.39 | 1.00 | 10.00 |  | 5.21 | 2.38 | 1.00 | 10.00 | 0.993 | 0.321 |
| PQ13 | 6.76 | 2.33 | 1.00 | 10.00 |  | 6.60 | 2.39 | 1.00 | 10.00 |  | 6.65 | 2.37 | 1.00 | 10.00 | 1.300 | 0.194 |
| PQ14 | 7.27 | 2.23 | 1.00 | 10.00 |  | 7.17 | 2.30 | 1.00 | 10.00 |  | 7.20 | 2.28 | 1.00 | 10.00 | 0.859 | 0.390 |
| PQ15 | 6.51 | 2.21 | 1.00 | 10.00 |  | 6.54 | 2.13 | 1.00 | 10.00 |  | 6.53 | 2.15 | 1.00 | 10.00 | 0.228 | 0.820 |
| PQ16 | 6.47 | 2.19 | 1.00 | 10.00 |  | 6.52 | 2.18 | 1.00 | 10.00 |  | 6.50 | 2.18 | 1.00 | 10.00 | 0.445 | 0.657 |
| PQ17 | 6.46 | 2.25 | 1.00 | 10.00 |  | 6.47 | 2.24 | 1.00 | 10.00 |  | 6.47 | 2.24 | 1.00 | 10.00 | 0.068 | 0.946 |
| PE1 | 6.92 | 2.28 | 1.00 | 10.00 |  | 6.97 | 2.31 | 1.00 | 10.00 |  | 6.96 | 2.30 | 1.00 | 10.00 | 0.436 | 0.663 |
| PE2 | 7.11 | 2.23 | 1.00 | 10.00 |  | 7.11 | 2.20 | 1.00 | 10.00 |  | 7.11 | 2.21 | 1.00 | 10.00 | 0.006 | 0.995 |

**Supplementary Table2 The measurement scores and comparative analysis between two groups of the initial draft of SIM_URRBMI (continued)**

| **Variables** | **Measurement variable selection group（*n*=574）** | | | |  | **Model evaluation group （*n*=1335）** | | | |  | **All（*n*=1909）** | | | | **Statistics** | ***P*** |
| --- | --- | --- | --- | --- | --- | --- | --- | --- | --- | --- | --- | --- | --- | --- | --- | --- |
|  | **$\overline{X}$** | **$S$** | **$X_{min}$** | **$X_{\max}$** |  | **$\overline{X}$** | **$S$** | **$X_{min}$** | **$X_{\max}$** |  | **$\overline{X}$** | **$S$** | **$X_{min}$** | **$X_{\max}$** |  |  |
| PE3 | 6.93 | 2.22 | 1.00 | 10.00 |  | 6.89 | 2.24 | 1.00 | 10.00 |  | 6.90 | 2.23 | 1.00 | 10.00 | 0.304 | 0.762 |
| PV1 | 6.31 | 2.16 | 1.00 | 10.00 |  | 6.35 | 2.11 | 1.00 | 10.00 |  | 6.33 | 2.13 | 1.00 | 10.00 | 0.330 | 0.741 |
| PV2 | 6.16 | 2.17 | 1.00 | 10.00 |  | 6.26 | 2.10 | 1.00 | 10.00 |  | 6.23 | 2.12 | 1.00 | 10.00 | 0.983 | 0.326 |
| PS1 | 6.25 | 2.17 | 1.00 | 10.00 |  | 6.34 | 2.14 | 1.00 | 10.00 |  | 6.31 | 2.15 | 1.00 | 10.00 | 0.792 | 0.428 |
| PS2 | 6.05 | 2.18 | 1.00 | 10.00 |  | 6.14 | 2.14 | 1.00 | 10.00 |  | 6.11 | 2.15 | 1.00 | 10.00 | 0.804 | 0.422 |
| PS3 | 6.15 | 2.20 | 1.00 | 10.00 |  | 6.09 | 2.16 | 1.00 | 10.00 |  | 6.11 | 2.17 | 1.00 | 10.00 | 0.545 | 0.586 |
| PC1 | 0.23 | 0.42 | 0.00 | 1.00 |  | 0.27 | 0.44 | 0.00 | 1.00 |  | 0.25 | 0.44 | 0.00 | 1.00 | 2.492 | 0.114 |
| PC2 | 0.02 | 0.14 | 0.00 | 1.00 |  | 0.03 | 0.16 | 0.00 | 1.00 |  | 0.03 | 0.15 | 0.00 | 1.00 | 0.904 | 0.342 |
| PT1 | 6.89 | 2.35 | 1.00 | 10.00 |  | 6.77 | 2.42 | 1.00 | 10.00 |  | 6.81 | 2.40 | 1.00 | 10.00 | 1.002 | 0.317 |
| PT2 | 6.64 | 2.52 | 1.00 | 10.00 |  | 6.70 | 2.51 | 1.00 | 10.00 |  | 6.68 | 2.51 | 1.00 | 10.00 | -0.461 | 0.645 |

**Supplementary Table3 Item parameter estimation from the GRM**

| **Variables** | ***a***  ***（SE）*** | ***b1***  ***（SE）*** | ***b2***  ***（SE）*** | ***b3***  ***（SE）*** | ***b4***  ***（SE）*** | ***b5***  ***（SE）*** | ***b6***  ***（SE）*** | ***b7***  ***（SE）*** | ***b8***  ***（SE）*** | ***b9***  ***（SE）*** | **$\overline{I}$** |
| --- | --- | --- | --- | --- | --- | --- | --- | --- | --- | --- | --- |
| PQ1 | 1（0） | -4.110（0.256） | -3.766（0.219） | -2.995（0.168） | -2.503（0.145） | -1.104（0.109） | -0.371（0.103） | 0.27（0.104） | 1.167（0.114） | 1.644（0.125） | 0.289 |
| PQ2 | 1.281（0.12） | -2.433（0.168） | -1.895（0.144） | -1.391（0.131） | -0.96（0.121） | 0.175（0.111） | 0.825（0.115） | 1.545（0.129） | 2.366（0.159） | 2.88（0.189） | 0.465 |
| PQ3 | 1.493（0.125） | -3.285（0.22） | -2.577（0.172） | -1.78（0.148） | -1.158（0.131） | 0.177（0.119） | 0.927（0.125） | 1.531（0.137） | 2.37（0.168） | 2.943（0.196） | 0.599 |
| PQ4 | 2.139（0.162） | -6.134（0.456） | -5.127（0.326） | -4.131（0.256） | -3.144（0.207） | -1.32（0.154） | -0.34（0.144） | 0.428（0.147） | 1.622（0.168） | 2.412（0.189） | 1.125 |
| PQ5 | 1.995（0.146） | -4.685（0.318） | -3.95（0.261） | -3.163（0.212） | -2.447（0.183） | -0.656（0.143） | 0.307（0.14） | 0.886（0.146） | 2.095（0.171） | 2.885（0.204） | 0.975 |
| PQ6 | 1.874（0.145） | -4.491（0.297） | -3.87（0.247） | -2.965（0.197） | -2.112（0.165） | -0.358（0.135） | 0.807（0.138） | 1.541（0.154） | 2.801（0.2） | 3.492（0.237） | 0.874 |
| PQ7 | 1.683（0.14） | -3.578（0.239） | -2.918（0.2） | -2.146（0.166） | -1.51（0.148） | -0.015（0.127） | 1.186（0.136） | 1.779（0.15） | 2.944（0.188） | 3.775（0.234） | 0.731 |
| PQ8 | 1.982（0.159） | -4.27（0.29） | -3.552（0.232） | -2.592（0.191） | -2.1（0.173） | -0.319（0.138） | 0.735（0.143） | 1.334（0.153） | 2.452（0.192） | 3.146（0.233） | 0.956 |
| PQ9 | 2.862（0.217） | -5.08（0.34） | -3.796（0.26） | -2.736（0.229） | -1.981（0.212） | 0.229（0.18） | 1.729（0.197） | 2.541（0.226） | 3.688（0.27） | 5.037（0.359） | 1.599 |
| PQ10 | 2.911（0.247） | -4.844（0.363） | -3.777（0.291） | -2.528（0.239） | -1.733（0.212） | 0.467（0.183） | 1.693（0.201） | 2.616（0.241） | 4.192（0.317） | 5.207（0.396） | 1.678 |
| PQ11 | 2.684（0.226） | -4.649（0.34） | -3.514（0.271） | -2.512（0.227） | -1.654（0.197） | 0.429（0.172） | 1.743（0.195） | 2.601（0.225） | 3.721（0.283） | 4.768（0.347） | 1.478 |

**Supplementary Table3 Item parameter estimation from the GRM (continued)**

| **Variables** | ***a***  ***（SE）*** | ***b1***  ***（SE）*** | ***b2***  ***（SE）*** | ***b3***  ***（SE）*** | ***b4***  ***（SE）*** | ***b5***  ***（SE）*** | ***b6***  ***（SE）*** | ***b7***  ***（SE）*** | ***b8***  ***（SE）*** | ***b9***  ***（SE）*** | **$\overline{I}$** |
| --- | --- | --- | --- | --- | --- | --- | --- | --- | --- | --- | --- |
| PQ12 | 2.414（0.21） | -4.477（0.329） | -3.314（0.248） | -2.177（0.202） | -1.45（0.18） | 0.491（0.159） | 1.936（0.187） | 2.814（0.222） | 3.838（0.28） | 4.708（0.34） | 1.281 |
| PQ13 | 2.137（0.177） | -5.564（0.402） | -4.621（0.313） | -3.688（0.252） | -2.978（0.214） | -1.198（0.156） | -0.175（0.144） | 0.664（0.149） | 1.839（0.183） | 2.564（0.219） | 1.099 |
| PQ14 | 1.573（0.148） | -5.27（0.426） | -4.673（0.324） | -3.983（0.257） | -3.314（0.204） | -1.454（0.134） | -0.574（0.121） | -0.055（0.12） | 0.947（0.134） | 1.651（0.156） | 0.681 |
| PQ15 | 2.719（0.222） | -6.536（0.549） | -5.811（0.429） | -4.759（0.336） | -3.569（0.259） | -1.218（0.181） | 0.107（0.172） | 1.021（0.185） | 2.553（0.234） | 3.798（0.304） | 1.582 |
| PQ16 | 3.05（0.233） | -7.221（0.576） | -6.006（0.421） | -4.802（0.322） | -3.85（0.281） | -1.395（0.199） | 0.267（0.189） | 1.219（0.2） | 3.099（0.267） | 4.168（0.326） | 1.855 |
| PQ17 | 2.397（0.196） | -5.767（0.467） | -4.974（0.356） | -4.136（0.285） | -3.22（0.235） | -1.06（0.163） | 0.115（0.157） | 0.987（0.167） | 2.432（0.21） | 3.391（0.262） | 1.332 |
| PE1 | 1.349（0.137） | -4.214（0.278） | -4.023（0.26） | -3.243（0.195） | -2.571（0.164） | -1.133（0.119） | -0.392（0.112） | 0.264（0.113） | 1.369（0.136） | 2.022（0.162） | 0.517 |
| PE2 | 1.435（0.148） | -4.751（0.338） | -4.252（0.277） | -3.424（0.211） | -2.938（0.182） | -1.384（0.126） | -0.463（0.116） | 0.127（0.117） | 1.204（0.135） | 1.837（0.161） | 0.573 |
| PE3 | 1.435（0.146） | -4.68（0.326） | -4.028（0.258） | -3.514（0.213） | -2.729（0.174） | -1.162（0.123） | -0.353（0.115） | 0.275（0.118） | 1.44（0.141） | 2.169（0.175） | 0.573 |
| PV1 | 3.056（0.24） | -6.853（0.542） | -5.884（0.424） | -4.561（0.321） | -3.768（0.283） | -1.246（0.198） | 0.372（0.188） | 1.491（0.207） | 3.465（0.287） | 4.699（0.363） | 1.884 |
| PV2 | 2.756（0.232） | -6.09（0.486） | -5.234（0.392） | -4.144（0.306） | -3.184（0.258） | -0.895（0.181） | 0.592（0.175） | 1.885（0.203） | 3.547（0.287） | 4.470（0.368） | 1.607 |

**Supplementary Table3 Item parameter estimation from the GRM (continued)**

| **Variables** | ***a***  ***（SE）*** | ***b1***  ***（SE）*** | ***b2***  ***（SE）*** | ***b3***  ***（SE）*** | ***b4***  ***（SE）*** | ***b5***  ***（SE）*** | ***b6***  ***（SE）*** | ***b7***  ***（SE）*** | ***b8***  ***（SE）*** | ***b9***  ***（SE）*** | **$\overline{I}$** |
| --- | --- | --- | --- | --- | --- | --- | --- | --- | --- | --- | --- |
| PS1 | 3.969（0.294） | -8.106（0.605） | -6.791（0.481） | -5.625（0.399） | -4.284（0.332） | -1.68（0.243） | 0.317（0.234） | 1.963（0.26） | 4.475（0.356） | 6.360（0.488） | 2.666 |
| PS2 | 3.666（0.299） | -7.407（0.629） | -6.332（0.483） | -5.052（0.384） | -3.778（0.31） | -1.243（0.226） | 0.803（0.222） | 2.237（0.261） | 4.303（0.353） | 6.345（0.498） | 2.361 |
| PS3 | 1.885（0.16） | -4.853（0.344） | -4.145（0.273） | -3.275（0.215） | -2.535（0.188） | -0.663（0.137） | 0.781（0.14） | 1.572（0.158） | 2.736（0.209） | 3.196（0.246） | 0.871 |
| PC1 | -0.889（0.167） | 1.443（0.153） | — | — | — | — | — | — | — | — | 0.197 |
| PC2 | 0.351（0.419） | 3.979（0.37） | — | — | — | — | — | — | — | — | 0.005 |
| PT1 | 1.553（0.152） | -4.243（0.289） | -3.927（0.255） | -3.481（0.219） | -2.963（0.197） | -1.061（0.129） | -0.231（0.119） | 0.567（0.123） | 1.262（0.139） | 1.865（0.167） | 0.660 |
| PT2 | 1.905（0.142） | -4.224（0.264） | -3.841（0.241） | -3.252（0.201） | -2.729（0.186） | -1.075（0.141） | -0.208（0.135） | 0.491（0.138） | 1.655（0.157） | 2.139（0.176） | 0.923 |

**Supplementary Table4** **The result of the collinearity assessment in the revised SIM_URRBMI1.0**

| **Direct path** | ***VIF*** | **Direct path** | ***VIF*** |
| --- | --- | --- | --- |
| PE→PQ | — | PE→PQ_information | 1.000 |
| PE→PV | 1.473 | PE→PQ_service | 1.000 |
| PE→PS | 1.590 | PE→PQ_policy | 1.000 |
| PQ→PV | 1.473 | PE→PQ_institution | 1.000 |
| PQ→PS | 2.803 | PQ_overall→PQ | 1.777 |
| PV→PS | 2.896 | PQ_information→PQ | 1.848 |
| PS→PC | 1.000 | PQ_service→PQ | 2.613 |
| PS→PT | 1.000 | PQ_policy→PQ | 2.796 |
| PE→PQ_overall | 1.000 | PQ_institution→PQ | 3.002 |

**Supplementary Table5 The indirect effects in the revised SIM_URRBMI1.0**

| **Indirect path** | **Coefficient** | **SE** | ***t*** | ***P*** | **95%CI** | |
| --- | --- | --- | --- | --- | --- | --- |
|  |  |  |  |  | **Lower** | **Upper** |
| PE→PV | 0.384 | 0.018 | 21.235 | 0.000 | 0.351 | 0.422 |
| PE→PS | 0.514 | 0.021 | 24.705 | 0.000 | 0.473 | 0.556 |
| PE→PC | -0.142 | 0.013 | 10.963 | 0.000 | -0.168 | -0.116 |
| PE→PT | 0.371 | 0.023 | 16.142 | 0.000 | 0.322 | 0.409 |
| PQ→PS | 0.316 | 0.025 | 12.729 | 0.000 | 0.269 | 0.364 |
| PQ→PC | -0.179 | 0.016 | 10.870 | 0.000 | -0.210 | -0.143 |
| PQ→PT | 0.467 | 0.018 | 25.732 | 0.000 | 0.430 | 0.502 |
| PV→PC | -0.113 | 0.013 | 9.010 | 0.000 | -0.139 | -0.090 |
| PV→PT | 0.297 | 0.023 | 13.161 | 0.000 | 0.252 | 0.336 |

**Questionnaire**

Questionnaire number

**Satisfaction survey of urban and rural resident-based basic medical insurance scheme for pupil in Changsha**

Hello, dear parents!

The basic medical insurance for urban and rural residents in Changsha was implemented on April 1, 2011, which means that urban resident-based basic medical insurance and the new rural cooperative medical system have been fully integrated.

In order to understand the degree of satisfaction of basic medical insurance of urban and rural residents (referred to as URRBMI in the following) for primary school students in changsha as well as your views and suggestions, we organized this survey. The survey is non-commercial in nature, and no fees are charged. Participation is voluntary. Participation has nothing to do with students' performance in school.

Your answer will provide very important information for the research, we sincerely hope to get your support and cooperation! We assure you that all information you provide will be kept strictly confidential.

If you agree to participate, please sign the informed consent form below and fill in the questionnaire carefully. If you have any questions, please feel free to contact us. Thank you for your support!

Instructions: Please fill in the form according to your actual situation. The child in the questionnaire refers to the primary school student who brings the questionnaire back to you.

**Part one: Sociodemographic information**

**Fill in instructions: please choose the option that you think best fits your situation or fill in the content that best fits the line according to your actual situation.**

1. what are your role for the child：

① Father ② Mother ③ Grandpa/grandma ④ Other： _________________

2. How old are you: _________

3. What’s your gender：① Male ② Female

4. What’s your marital status：

① Married ② Divorced ③ Unmarried ④ Other： _________________

5. What’s your education level：

① Junior high school and below ② High school / vocational college ③junior college ④ Bachelor ⑤ Master degree or above

6. What’s your child gender： ① Male ② Female

7. How old are your child：_________

8. How is your child's health：

① Very good ② Good ③ General ④ Bad

9. Whether your child has been ill in the past one year?

① Yes ② No

10. What are your child's non-deductible medical expenses (yuan) in the past one year?

① <1000 ② 1001-2000 ③ 2001-3000 ④ 3001-4000 ⑤ >4000

**Part two: Satisfaction information**

**The two ends of the scale below represent two different attitudes towards the same question. The higher the score you tick, the more you agree with the attitude on the right side of the scale. The smaller the score you tick, the more you agree with the attitude to the left of the scale. Please check the score you think best expresses your idea according to your actual feeling.**

PQ1. Think back your child's insurance participation experience in the past 1 year, how would you rate your overall evaluation of the quality of URRBMI? Please use a 10 point scale on which “1” means “very bad” and “10” means “very good”.

|  |  |  |  |  |  |  |  |  |  |  |  |
| --- | --- | --- | --- | --- | --- | --- | --- | --- | --- | --- | --- |

Very bad 1 2 3 4 5 6 7 8 9 10 Very good

PQ2. Using a 10 point scale on which “1” means “very little” and “10” means “very much”, to what extent can you obtain the relevant information required for your child to participate in the basic medical insurance?

|  |  |  |  |  |  |  |  |  |  |  |  |
| --- | --- | --- | --- | --- | --- | --- | --- | --- | --- | --- | --- |

Very little 1 2 3 4 5 6 7 8 9 10 Very much

PQ3. Using a 10 point scale on which “1” means “very difficult” and “10” means “very convenient”, how convenient is it for you to obtain information about your child's basic medical insurance?

|  |  |  |  |  |  |  |  |  |  |  |  |
| --- | --- | --- | --- | --- | --- | --- | --- | --- | --- | --- | --- |

Very difficult 1 2 3 4 5 6 7 8 9 10 Very convenient

PQ4. Using a 10 point scale on which “1” means “very bad” and “10” means “very good”, how would you rate the attitude of the staff when you accept URRBMI service?

|  |  |  |  |  |  |  |  |  |  |  |  |
| --- | --- | --- | --- | --- | --- | --- | --- | --- | --- | --- | --- |

Very bad 1 2 3 4 5 6 7 8 9 10 Very good

PQ5. Using a 10 point scale on which “1” means “very unclear” and “10” means “very clear”, how clear are the explanations of relevant policies when you receive URRBMI service ?

|  |  |  |  |  |  |  |  |  |  |  |  |
| --- | --- | --- | --- | --- | --- | --- | --- | --- | --- | --- | --- |

Very unclear 1 2 3 4 5 6 7 8 9 10 Very clear

PQ6. Using a 10 point scale on which “1” means “completely unsatisfied” and “10” means “completely satisfied”, to what extent did the URRBMI meet your child's basic medical insurance needs?

|  |  |  |  |  |  |  |  |  |  |  |  |
| --- | --- | --- | --- | --- | --- | --- | --- | --- | --- | --- | --- |

Completely unsatisfied 1 2 3 4 5 6 7 8 9 10 Completely satisfied

PQ7. Using a 10 point scale on which “1” means “completely unsatisfied” and “10” means “completely satisfied”, to what extent does URRBMI meet your child's personalized medical insurance needs (such as outpatient service for special diseases, etc.)

|  |  |  |  |  |  |  |  |  |  |  |  |
| --- | --- | --- | --- | --- | --- | --- | --- | --- | --- | --- | --- |

Completely unsatisfied 1 2 3 4 5 6 7 8 9 10 Completely satisfied

PQ8. Using a 10 point scale on which “1” means “completely unsatisfied” and “10” means “completely satisfied”, how satisfied are you with the payment level of URRBMI?

|  |  |  |  |  |  |  |  |  |  |  |  |
| --- | --- | --- | --- | --- | --- | --- | --- | --- | --- | --- | --- |

Completely unsatisfied 1 2 3 4 5 6 7 8 9 10 Completely satisfied

PQ9. Using a 10 point scale on which “1” means “completely unsatisfied” and “10” means “completely satisfied”, how satisfied are you with the scope of reimbursement of URRBMI?

|  |  |  |  |  |  |  |  |  |  |  |  |
| --- | --- | --- | --- | --- | --- | --- | --- | --- | --- | --- | --- |

Completely unsatisfied 1 2 3 4 5 6 7 8 9 10 Completely satisfied

PQ10. Using a 10 point scale on which “1” means “completely unsatisfied” and “10” means “completely satisfied”, how satisfied are you with the reimbursement proportion of URRBMI?

|  |  |  |  |  |  |  |  |  |  |  |  |
| --- | --- | --- | --- | --- | --- | --- | --- | --- | --- | --- | --- |

Completely unsatisfied 1 2 3 4 5 6 7 8 9 10 Completely satisfied

PQ11. Using a 10 point scale on which “1” means “completely unsatisfied” and “10” means “completely satisfied”, how satisfied are you with the deductible level of URRBMI?

|  |  |  |  |  |  |  |  |  |  |  |  |
| --- | --- | --- | --- | --- | --- | --- | --- | --- | --- | --- | --- |

Completely unsatisfied 1 2 3 4 5 6 7 8 9 10 Completely satisfied

PQ12. Using a 10 point scale on which “1” means “completely unsatisfied” and “10” means “completely satisfied”, how satisfied are you with the capitation of URRBMI?

|  |  |  |  |  |  |  |  |  |  |  |  |
| --- | --- | --- | --- | --- | --- | --- | --- | --- | --- | --- | --- |

Completely unsatisfied 1 2 3 4 5 6 7 8 9 10 Completely satisfied

PQ13. Using a 10 point scale on which “1” means “very bad” and “10” means “very good ”, how would you rate the feeling about the service quality of community or village committees for enrollment when you insured your child for URRBMI?

|  |  |  |  |  |  |  |  |  |  |  |  |
| --- | --- | --- | --- | --- | --- | --- | --- | --- | --- | --- | --- |

Very bad 1 2 3 4 5 6 7 8 9 10 Very good

PQ14. Using a 10 point scale on which “1” means “very bad” and “10” means “very good ”, how would you rate the feeling about the quality of payment bank or online payment platform when you insured your child for URRBMI?

|  |  |  |  |  |  |  |  |  |  |  |  |
| --- | --- | --- | --- | --- | --- | --- | --- | --- | --- | --- | --- |

Very bad 1 2 3 4 5 6 7 8 9 10 Very good

PQ15. Using a 10 point scale on which “1” means “very bad” and “10” means “very good ”, how would you rate the feeling about the service quality of authorized medical institution?

|  |  |  |  |  |  |  |  |  |  |  |  |
| --- | --- | --- | --- | --- | --- | --- | --- | --- | --- | --- | --- |

Very bad 1 2 3 4 5 6 7 8 9 10 Very good

PQ16. Using a 10 point scale on which “1” means “very bad” and “10” means “very good ”, how would you rate the feeling about the service quality of reimbursement in medical institution?

|  |  |  |  |  |  |  |  |  |  |  |  |
| --- | --- | --- | --- | --- | --- | --- | --- | --- | --- | --- | --- |

Very bad 1 2 3 4 5 6 7 8 9 10 Very good

PQ17. Using a 10 point scale on which “1” means “very bad” and “10” means “very good”, how would you rate the feeling about the service quality of authorized pharmacy?

|  |  |  |  |  |  |  |  |  |  |  |  |
| --- | --- | --- | --- | --- | --- | --- | --- | --- | --- | --- | --- |

Very bad 1 2 3 4 5 6 7 8 9 10 Very good

PE1. Before you buying insurance for your child, you may already know something about the policy. Now think back and remember your expectations of the overall quality of URRBMI. Using a 10 point scale on which “1” means your expectations were “very low” and “10” means your expectations were “very high”, how would you rate your expectations of the overall quality of URRBMI?

|  |  |  |  |  |  |  |  |  |  |  |  |
| --- | --- | --- | --- | --- | --- | --- | --- | --- | --- | --- | --- |

Very low 1 2 3 4 5 6 7 8 9 10 Very high

PE2. Before you buying insurance for your child, you may already know something about the policy. Using a 10 point scale on which “1” means your expectations were “very low” and “10” means your expectations were “very high” how would you rate your expectations of URRBMI to ensure basic medical needs?

|  |  |  |  |  |  |  |  |  |  |  |  |
| --- | --- | --- | --- | --- | --- | --- | --- | --- | --- | --- | --- |

Very low 1 2 3 4 5 6 7 8 9 10 Very high

PE3. Before you buying insurance for your child, you may already know something about the policy. Considering your child's health, how would you rate your expectations of URRBMI to ensure personalization medical needs (Such as special disease clinic)? Please use a 10 point scale on which “1” means your expectations were “very low” and “10” means your expectations were “very high”.

|  |  |  |  |  |  |  |  |  |  |  |  |
| --- | --- | --- | --- | --- | --- | --- | --- | --- | --- | --- | --- |

Very low 1 2 3 4 5 6 7 8 9 10 Very high

PV1. According to the current payment level, how would you rate the quality of URRBMI? Please use a 10 point scale on which “1” means “very bad” and “10” means “very good”.

|  |  |  |  |  |  |  |  |  |  |  |  |
| --- | --- | --- | --- | --- | --- | --- | --- | --- | --- | --- | --- |

Very bad 1 2 3 4 5 6 7 8 9 10 Very good

PV2. According to the current quality of medical insurance, how would you rate the premium level of URRBMI? Please use a 10 point scale on which “1” means “very bad” and “10” means “very good”.

|  |  |  |  |  |  |  |  |  |  |  |  |
| --- | --- | --- | --- | --- | --- | --- | --- | --- | --- | --- | --- |

Very bad 1 2 3 4 5 6 7 8 9 10 Very good

PS1. Combined with your child's current participation and reimbursement experience, how would you rate your overall satisfaction with URRBMI? Please use a 10 point scale on which “1” means “completely unsatisfied” and “10” means “completely unsatisfied”.

|  |  |  |  |  |  |  |  |  |  |  |  |
| --- | --- | --- | --- | --- | --- | --- | --- | --- | --- | --- | --- |

Completely unsatisfied 1 2 3 4 5 6 7 8 9 10 Completely satisfied

PS2. Compared with previous expectations, how satisfied are you with the current status of services for URRBMI? Please use a 10 point scale on which “1” means “completely unsatisfied” and “10” means “completely unsatisfied”.

|  |  |  |  |  |  |  |  |  |  |  |  |
| --- | --- | --- | --- | --- | --- | --- | --- | --- | --- | --- | --- |

Completely unsatisfied 1 2 3 4 5 6 7 8 9 10 Completely satisfied

PS3. Suppose there is a kind of ideal primary school student basic medical insurance, how well do you think the URRBMI compares with that ideal primary school student basic medical insurance? Please use a 10 point scale on which “1” means “far to the ideal” and “10” means “close to the ideal”.

|  |  |  |  |  |  |  |  |  |  |  |  |
| --- | --- | --- | --- | --- | --- | --- | --- | --- | --- | --- | --- |

Far 1 2 3 4 5 6 7 8 9 10 Close

PC1. In the past year, have you complained informally about URRBMI?

A Yes B Not C Unknown

PC2. In the past year, have you complained formally about URRBMI?

A Yes B Not C Unknown

PT1. Using a 10 point scale on which “1” means “very unwilling” and “10” means “very willing”, how would you rate your willing to make a positive evaluation of URRBMI?

|  |  |  |  |  |  |  |  |  |  |  |  |
| --- | --- | --- | --- | --- | --- | --- | --- | --- | --- | --- | --- |

Very unwilling 1 2 3 4 5 6 7 8 9 10 Very willing

PT2. Using a 10 point scale on which “1” means “very unlikely” and “10” means “very likely”, how likely is it that you will recommend URRBMI to others?

|  |  |  |  |  |  |  |  |  |  |  |  |
| --- | --- | --- | --- | --- | --- | --- | --- | --- | --- | --- | --- |

Very unlikely 1 2 3 4 5 6 7 8 9 10 Very likely

Thank you for your support and cooperation!
